# Estimating the Economic Impact of Levalbuterol’s Potential Transition From the National Reimbursement Drug List for the Treatment of Pediatric Asthma in China: A Budget Impact Analysis

**DOI:** 10.7759/cureus.60640

**Published:** 2024-05-19

**Authors:** Tingke Tang, Chunlong Lin, Canghong Zhi, Xuan Li, Yingyu Wu

**Affiliations:** 1 Department of Pharmacoeconomics, School of International Pharmaceutical Business, China Pharmaceutical University, Nanjing, CHN; 2 Department of Respiratory and Critical Care Medicine, Yueyang People's Hospital Affiliated to Hunan Normal University, Yueyang, CHN; 3 Department of Medical Affair, Joincare Pharmaceutical Group Industry Co. Ltd., Shenzhen, CHN

**Keywords:** children, levalbuterol, china, budget impact analysis, asthma

## Abstract

Background

Levalbuterol is a short-acting β_2_-agonist (SABA) indicated for treating or preventing asthma exacerbation. It was included in the 2020 Chinese National Reimbursement Drug List (NRDL). This study estimates the economic impact of levalbuterol’s status change within and withdrawal from the NRDL in treating pediatric asthma from a publicly funded medical insurance perspective.

Methodology

A prevalence-based budget impact model was developed. The analysis compared a *world with* a levalbuterol scenario to a *world without* levalbuterol. Epidemiological data were obtained from the existing literature. Cost data were estimated based on the drug dosage in clinical trials, real-world settings, and expert opinions. Scenario analysis considered the same length of stay (LOS) in the two groups. One-way sensitivity analyses were carried out to show the impact of varying individual parameters.

Results

In the base-case analysis, compared to the *world without* scenario, the preservation of levalbuterol resulted in cost savings of ¥82.8 million in China over three years. In the scenario analysis, savings decreased to ¥76.1 million over three years. Sensitivity analysis showed that, for the most part, the results were robust to changes in input parameter values.

Conclusions

Using levalbuterol may lead to substantial cost savings for Chinese society.

## Introduction

Asthma is one of the most prevalent chronic diseases in the pediatric population in China. In 2010, the estimated prevalence of asthma was 3.02% in Chinese children aged 0 to 14 years and is increasing [1]. Approximately 77% of asthmatic children will suffer from at least one exacerbation, among which 47.3% will be admitted to the hospital [2]. Per child averaged over the hospitalization period, asthma-related costs were estimated as 2,836 to 3,856 yuan [2]. Overall, the total direct medical cost of pediatric asthma is estimated to be over ¥200 billion (US$30.56 billion) in healthcare costs each year. Asthma exacerbation not only seriously affects the quality of life of children with asthma but also greatly burdens families and society, related to morbidity and mortality [3]. It is estimated that approximately 15 million daily adjusted life years (DALYs) are lost each year, accounting for 1% of the global disease burden [4,5].

Current guidelines recommend that nebulized short-acting β_2_-agonist (SABA) therapy, such as albuterol, terbutaline, and levalbuterol, is the first-line therapy in children with acute asthma across all age groups for symptom relief [6,7]. However, higher SABA use has resulted in an increase in SABA-related adverse drug reactions (ADRs) with a higher likelihood of experiencing asthma exacerbations [6,7], emergency department visits and overnight hospital stays [6,7], and even mortality [6,7].

As a purified albuterol, (R)-albuterol (levalbuterol) has been proven to be clinically comparable to four- to eight-fold higher doses of albuterol [8]. There are several randomized clinical trials of levalbuterol, and both their results and the real-world data indicate that, compared with other SABAs, levalbuterol seems to be more effective and safer, with a lower risk of hospitalization, emergency department visits, and adverse events in children [9-11]. Considering its cost-effectiveness, levalbuterol has been included in China's National Reimbursement Drug List (NRDL) since 2020. However, the economic impact of using levalbuterol for the treatment of asthma in children in China has not been studied.

The purpose of this study was to analyze the economic effects of levalbuterol’s status change within and withdrawal from the NRDL, as a treatment for asthma in children, affecting the budget of the Chinese healthcare payer.

We present the following article by the Consolidated Health Economic Evaluation Reporting Standards (CHEERS) reporting checklist (Appendix) [12].

## Materials and methods

Model description and structure

**Figure 1:**
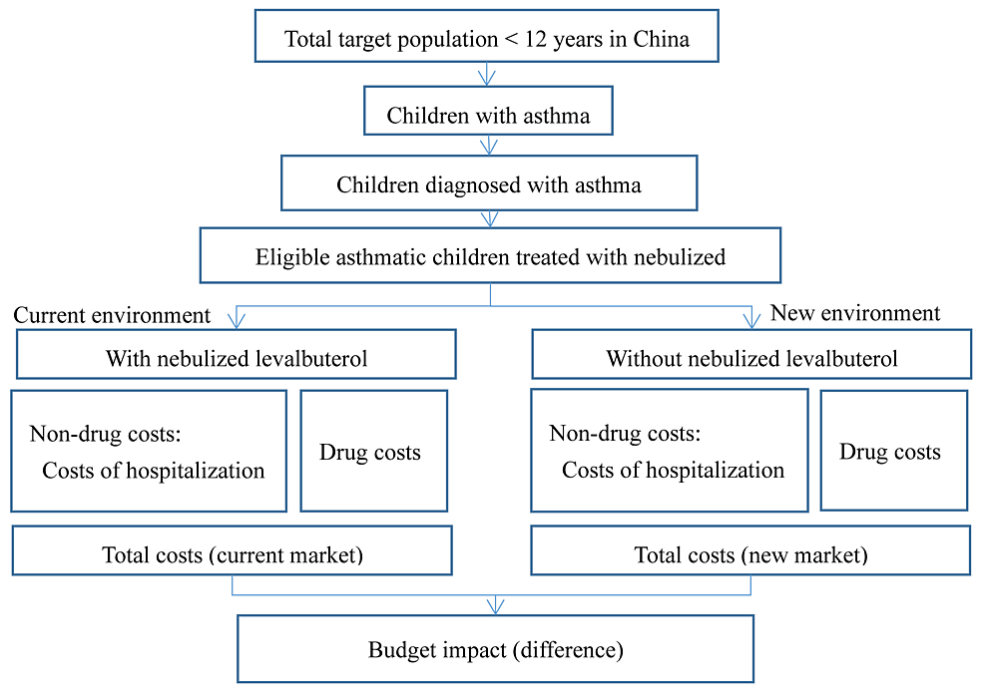
Budget impact model structure. Source: [13].

We used Microsoft Excel 2016 to build a three-year budget impact model (BIM), assessing levalbuterol’s economic outcomes within the NRDL for pediatric asthma patients under 12. Figure 1 depicts a schematic representation of the model [13]. Incorporated within this model structure are the prevalence of asthma, the allocation of market shares among various medications, the proportion of medication usage, and the hospitalization costs in China. The model's ethical review was not required as it involved no personal patient data.

The analysis compared a *world with* a levalbuterol scenario to a *world without* a levalbuterol scenario from the public-funded medical insurance perspective and included only direct medical costs. Direct medical costs include pharmacy costs and hospitalization costs. Discounting was not considered in this study, consistent with recommendations of the International Society for Pharmacoeconomics and Outcomes Research (ISPOR) [14].

In scenario analysis, we hypothesized that the length of stay (LOS) remained consistent across both cohorts, averaging 8.03 days, in light of the contradictory results about the advantages of the levalbuterol group as compared to the albuterol and terbutaline group. Total healthcare expenditures were calculated using a macro-costing approach, aggregating resource utilization and applying unit costs in renminbi (RMB, ¥) for 2022.

Model inputs and assumptions

Population

The population parameters are shown in Table 1.

**Table 1:** Population parameters. SABA, short-acting β_2_ agonist

Parameters	Data	Source
Natural population growth rate	0.46%	National Bureau of Statistics [15-18]
Overall population in China in 2022	1,413,080,284	National Bureau of Statistics [15-18], calculated based on population in 2020 and growth rate
Proportion of children under 12 years old	13.11%	National Bureau of Statistics [18]
Rate of insurance coverage	97%	National Bureau of Statistics [18]
Acute asthma attack rate	61.45%	National Cooperative Group on Childhood et al. [19]
Prevalence rate of asthma	4.91%	National Cooperative Group on Childhood et al. [19]
Asthma diagnosis rate	70.70%	National Cooperative Group on Childhood et al. [19]
Proportion of children treated with nebulized SABAs	73.4%	National Cooperative Group on Childhood et al. [19]

Data from the National Bureau of Statistics, including the natural population growth rate, the overall population, the proportion of children under 12 years of age, and the rate of insurance coverage, were used to estimate the number of children under 12 years of age across Medicare plans [15-18]. The asthma diagnosis rate, acute asthma attack rate, and proportion of children treated with nebulized SABAs were derived from a cross-sectional epidemiological trial conducted in 43 cities among the general population of children [19]. The prevalence of asthma was based on the same epidemiological trial, and the expert panel provided the final statistics. This expert panel, which reflected actual clinical practice, was made up of 70 important chief doctors or prominent physicians with expertise in pediatric respiratory diseases who were chosen from China's most representative institutions. These experts engaged in a comprehensive survey, followed by a Delphi process involving repetitive rounds of discussions and systematic queries to reach a collective agreement.

Treatment Comparators and Market Share

Comparators selected for BIM were based on the most recent Recommendations for the Diagnosis and Management of Bronchial Asthma in Children (2020) for the management of acute asthma [6]. Comparators included nebulized albuterol, terbutaline, and levalbuterol (Table 2).

**Table 2:** Budget impact model comparators.

Comparators	Dosing	Asthma treatment duration
Albuterol Sulfate Nebulized Inhalation Solution 2.5 mL: 2.5 mg	2.5 mg, four times per day	Outpatient: 3 days; Resident: 7 days
Albuterol Sulfate Nebulized Inhalation Solution 2.5 mL: 5 mg	2.5 mg, four times per day	Outpatient: 3 days; Resident: 7 days
Albuterol Sulfate Nebulized Inhalation Solution 2 mL: 10 mg	2.5 mg, four times per day	Outpatient: 3 days; Resident: 7 days
Terbutaline Sulfate Solution for Nebulization 2 mL: 5 mg	2.5 mg, four times per day	Outpatient: 3 days; Resident: 7 days
Levalbuterol Hydrochloride Inhalation Solution 3 mL: 0.31 mg	Low dose: 0.31 mg, high dose: 0.63 mg, dose frequency: four times per day	Outpatient: 3 days; Resident: 7 days

The data about the treatment course in both outpatient and hospitalized settings in pediatric asthma were obtained from the expert panel. Table 3 illustrates the distribution of market shares among comparator drugs in a scenario where levalbuterol is absent from the current market. Data from January 2015 to December 2022 in the information management system (IMS) was used to calculate market shares in both the new and present settings.

**Table 3:** Market shares and average wholesale price applied in the budget impact model. Market shares in the current and new environments were based on data on file: (1) In the current environment, levalbuterol is listed on the National Reimbursement Drug List (NRDL), but Lishutong’s market share is slowly declining due to the popularity of volume-based procurement for Salbutamol and Terbutaline, as well as the availability of other Levalbuterol generics. (2) In the new environment, Levalbuterol is removed from the NRDL, and the market share of the Lishutong and other levalbuterol counterparts will decrease. Salbutamol and terbutaline, on the other hand, are experiencing an annual increase in market share due to their popularity in VBP. The market share of these drugs not purchased through VBP is declining each year. Source of AWP: DRUGDATAEXPY Website (https://db.yaozh.com). AWP, average wholesale price; VBP, volume-based procurement

Comparator	Current environment (%)			New environment (%)		
	Year 1	Year 2	Year 3	Year 1	Year 2	Year 3
Lishutong	4.48%	3.85%	3.17%	3.41%	2.42%	1.72%
Other levalbuterol	4.14%	5.93%	7.47%	2.17%	2.14%	2.11%
Abuterol-VBP	16.05%	18.49%	20.28%	14.97%	16.80%	18.30%
Abuterol-non-VBP	11.68%	10.69%	9.78%	11.75%	10.81%	9.94%
Terbutaline-VBP	18.14%	28.05%	35.37%	13.73%	21.42%	28.00%
Terbutaline-non-VBP	45.50%	32.99%	23.92%	53.97%	46.42%	39.92%

The BIM was deliberately designed to reflect market share acquisition for levalbuterol at the expense of albuterol, as the latter served as the comparative agent in the referenced randomized controlled trial (RCT) [8]. Considering the influences of volume-based procurement (VBP) policy, we predicted a slight decrease in the market share of levalbuterol (while within the NRDL) over the next three years. If levalbuterol was withdrawn from the NRDL, the market share of levalbuterol would decline further.

Assumptions and Clinical Inputs

An electronic search was conducted in the following databases: Medline, Embase, Cochrane Library, Chinese Biologic Medical Literature (CBM), Chinese National Knowledge Infrastructure (CNKI), Chongqing VIP, and Chinese WanFang, for studies published up to May 6, 2023, using the keywords "levalbuterol, levosalbutamol, albuterol, levalbuterol, and terbutaline." The following standards were used to determine whether clinical studies were eligible: (1) studies with an RCT design; (2) participants consisting of children and adolescents aged 18 years or younger; (3) diagnosis of asthma established by a physician using suitable diagnostic benchmarks, which encompasses children under one year exhibiting wheezing symptoms; (4) comparing levalbuterol with albuterol or terbutaline (all of the medications restricted to the inhaled solution), without limitations on the treatment duration, frequency of administration, or dosage. There was no limitation of language.

The preliminary search yielded 63 references, with an additional three references found. After removing duplicates, 60 references remained. Nineteen records were included after the first-level screening's titles and abstracts were assessed. Subsequently, a secondary, full-text evaluation led to the exclusion of 12 further records. A total of seven RCTs comparing levalbuterol and albuterol were included. No RCT that met the exclusion criteria was included. Among the above seven RCTs, the hospital admission rate and the change in pulmonary function were reported in one and three trials, respectively. All trials reported adverse events. Only one trial favored levalbuterol for reducing the risk of hospitalization. One study reported a similar improvement in pulmonary function between 0.31 mg levalbuterol and 2.5 mg albuterol. Another trial reported a similar improvement in pulmonary function between 0.31 mg levalbuterol and 1.25 mg albuterol. Only one study reported a clinical benefit of levalbuterol in reducing the risk of rapid heart rate and hypokalemia. The other five studies reported a similar safety effect between levalbuterol and albuterol.

In this study, levalbuterol and other inhaled SABAs were considered to have equivalent efficacy and safety due to the heterogeneous quality and conflicting results of the published studies. The dosage and frequency of administration of levalbuterol, albuterol, and terbutaline were derived from the package insert.

In the base-case scenario, the assumption was that levalbuterol was clinically comparable to eightfold higher doses of albuterol with more safety advantages based on a published RCT [8], while the efficacy and safety were assumed to be equivalent between albuterol and terbutaline. All of the clinical parameters are shown in Table 4.

**Table 4:** All clinical and cost parameters. *Source of drug price: DRUGDATAEXPY Website (https://db.yaozh.com).

Parameters	Data					Source
	Levalbuterol		Albuterol		Terbutaline	
Base-case analysis						
Annual asthma-associated hospitalization rate	1.1					Experts panel
Levalbuterol: length of hospitalization (day)	7.17					Carl et al. [9]
Albuterol: length of hospitalization (day)	8.03					Carl et al. [9]
Levalbuterol: hospitalization costs per visit (without drug)	¥1226.77					Chen et al. [20], Zhou et al. [21], Wang et al. [22]
Albuterol & terbutaline: hospitalization costs per visit (without drug)	¥1373.91					Chen et al. [20], Zhou et al. [21], Wang et al. [22]
Scenario analysis						
Rate of hospitalization	45%	45%		45%		Carl et al. [9]
Levalbuterol: hospitalization costs per visit (without drug)	¥1373.91					Chen et al. [20], Zhou et al. [21], Wang et al. [22]
Albuterol and terbutaline: hospitalization costs per visit (without drug)	¥1373.91					Chen et al. [20], Zhou et al. [21], Wang et al. [22]
Other parameters						
Reimbursement ratio in an outpatient setting	50%					National Bureau of Statistics
Reimbursement ratio in a resident setting	65%					National Bureau of Statistics
Levalbuterol: Daily dose (mg)	0.93					Package insert
Albuterol and terbutaline: Daily dose (mg)	5					Package insert
Levalbuterol, per 1 mg (unit price)	¥23.11					Calculated based on price* and market share in 2022
Albuterol-VBP, per 1 mg (unit price)	¥0.47					
Albuterol-non-VBP, per 1 mg (unit price)	¥3.99					
Terbutaline-VBP, per 1 mg (unit price)	¥0.27					
Terbutaline-non-VBP, per 1 mg (unit price)	¥1.89					

Cost Inputs

This BIM only covered the direct expenses of healthcare. Drug expenditures and treatment-related medical expenses were included in the category of direct medical costs. Drug costs were obtained from the Drugdataexpy website and were based on average wholesale prices (AWPs) (Table 4).

The final costs of hospitalization applied for BIM were obtained from the expert panel based on published studies [20-22]. Nonmedical and indirect costs associated with caregiving and lost productivity, as well as additional nutrition costs, were not included in the BIM. All cost parameters are presented as 2022 renminbi in Table 4.

Model outputs for the base-case model: In the settings with and without levalbuterol, total expenses, as well as aggregated costs per budget category (pharmacy and medical), were ascertained. Calculations were made by multiplying the size of the target population by the projected market share of each comparator, followed by multiplication with the respective pharmacy and medical treatment costs. The projected financial impact of introducing levalbuterol (total incremental cost) was calculated from the variance in total costs between the existing and new market conditions.

Statistical analysis

Scenario Analysis

Given the contradictory results on the advantages of levalbuterol in comparison to albuterol, we presumed that the length of hospital stay was comparable in the levalbuterol group.

One-Way Sensitivity Analysis

To evaluate the robustness of the model, a one-way sensitivity analysis was performed on the base case model for certain inputs. The sensitivity analysis examined the key factors influencing the results of the budget impact by altering each input's base-case value independently (Table 5).

**Table 5:** Range of input parameters in a sensitivity analysis.

Input parameters	Base-case setting	Low input	High input
Prevalence rate of asthma (%)	6.59	5.93	7.25
Asthma visit rate (%)	52.70	47.43	57.97
Asthma diagnosis rate (%)	70.7	60.7	80.7
Proportion of children treated with nebulized SABAs (%)	66.15	59.54	72.77
Total market share of levalbuterol (%)	4.81	4.33	5.29
AWP of levalbuterol (¥/mg)	23.11	20.80	25.42
Length of hospital stay: levalbuterol (days)	7.17	6.45	7.89
Length of hospital stay: albuterol and terbutaline (days)	8.03	7.23	8.83

## Results

The target population of children with asthma under 12 years of age treated with nebulized SABAs in the three-year horizon was estimated to be 6.87 million, while those covered by national medical insurance were estimated to be 6.63 million in China (Table 6).

**Table 6:** Base-case analysis results of BIM in the current and new environments. BIM, budget impact model

	Year 1	Year 2	Year 3	Total
Total target population (million)	2.29	2.29	2.29	6.87
Total direct costs				
Current environment (¥, million)	4,449.81	4,402.61	4,366.94	13,219.37
Drug costs (¥, million)	392.46	350.82	319.28	1,062.57
Hospitalization costs (¥, million)	4,057.35	4,051.79	4,047.66	12,156.80
New environment (¥, million)	4,469.33	4,432.30	4,400.50	13,302.13
Drug costs (¥, million)	397.41	355.48	320.19	1,073.08
Hospitalization costs (¥, million)	4,071.92	4,076.82	4,080.31	12,229.05

Base-case analysis

**Figure 2:**
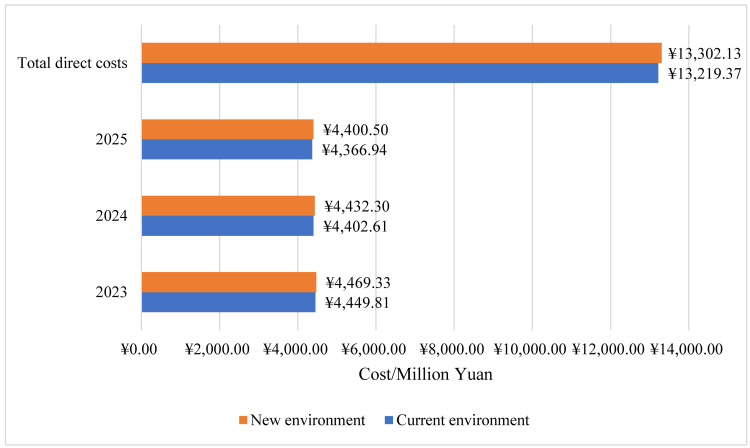
The total annual direct medical costs of current and new environments in pediatric asthma of the base-case analysis.

According to estimates, the combined yearly expenses, including drug and non-drug costs, amounted to ¥13,219.4 million for levalbuterol in the NDRL (current environment). As for the new environment (levalbuterol withdrawal from the NDRL), the total expenses gained to ¥13,302.1 million, resulting in a budget depletion of ¥82.8 million over three years (Figure 2; Table 6).

Scenario and sensitivity analysis

A scenario analysis was performed under the assumption that patients receiving levalbuterol treatment have a similar duration of hospitalization to those receiving albuterol or terbutaline treatment. The total annual costs (drug and nondrug costs) were estimated to be ¥13,345.30 million for levalbuterol in the NDRL (current environment) compared to ¥13,362.69 million for levalbuterol withdrawal from the NDRL (new environment), leading to a total budget depleting of ¥17.39 million after three years (Table 7).

**Table 7:** Scenario analysis results of BIM considering fewer hospitalization events in the current and new environments (millions). BIM, budget impact model

	Year 1	Year 2	Year 3	Total
Total target population (million)	2.29	2.29	2.29	6.87
Total direct costs				
Current environment (¥, million)	4,487.19	4,445.02	4,413.09	13,345.30
Drug costs (¥, million)	392.46	350.82	319.28	1,062.57
Hospitalization costs (¥, million)	4,094.73	4,094.20	4,093.80	12,282.73
New environment (¥, million)	4,493.53	4,452.06	4,417.10	13,362.69
Drug costs (¥, million)	397.41	355.48	320.19	1,073.08
Hospitalization costs (¥, million)	4,096.11	4,096.58	4,096.91	12,289.61

**Figure 3:**
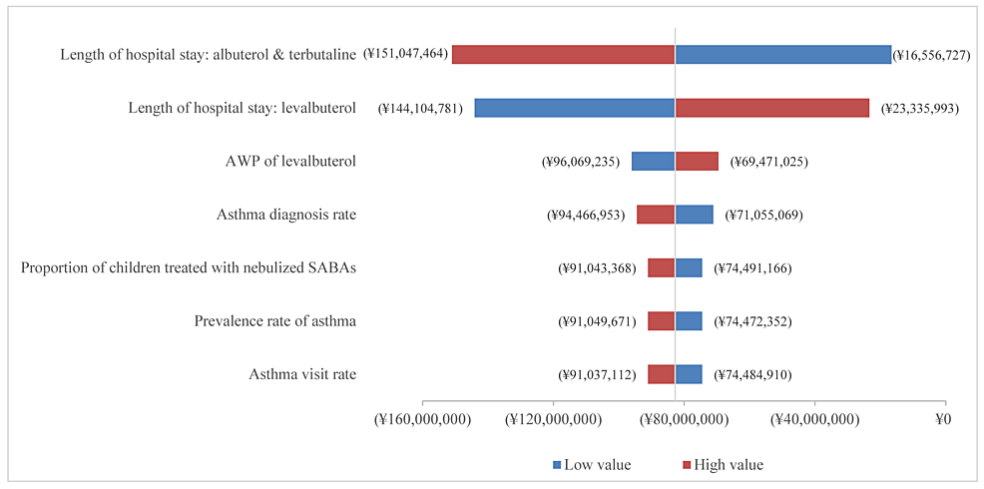
One-way sensitivity analysis tornado chart for the base-case analysis of budget impact. AWP, average wholesale price; SABA, short-acting β2 agonist

The results of the one-way sensitivity analysis showed that the LOS of albuterol and terbutaline group, the LOS of levalbuterol group, and AWPs of levalbuterol were found to have the largest impact on the model results in the base-case analysis (Figure 3).

## Discussion

The objective of this study was to estimate the economic impact of changes in levalbuterol's inclusion in the NRDL on pediatric asthma treatment costs. Findings indicate potential budget savings from the use of levalbuterol in asthmatic children under 12 years of age. This research offers supporting evidence, suggesting policymakers should consider integrating levalbuterol as an improved therapeutic option for managing pediatric asthma in China.

It is important to note that there is an ongoing debate concerning the clinical benefit of levalbuterol vs. albuterol because of the significant pharmacy cost difference between these two SABAs and insufficient evidence supporting the clinical benefits of levalbuterol. Previous studies have proven that levalbuterol has more safety and clinical benefits than albuterol. Without (S)-albuterol, a stimulatory factor of intracellular calcium accumulation and an inhibitory factor of adenyl cyclase [23], levalbuterol (R-) might be safer because of the lower risk of inflammation [24]. Moreover, levalbuterol is more effective than albuterol, which may be induced by its higher binding affinity to the β_2_-receptor (100-fold greater than (S)-albuterol) [25], resulting in longer bronchoprotective and bronchodilator effects (almost six to eight hours), which requires a smaller dose and less frequent dosing. A single-center, double-blind RCT, including 482 asthmatic patients aged 1 to 18 years, indicated that the hospitalization rate was significantly lower in the levalbuterol group than in the albuterol group (36% vs. 45%, *P *= 0.02) [9]. A meta-analysis, including seven RCTs, also supported the conclusion that levalbuterol has a lower risk of hospitalization admission (odds ratio [OR] 0.76; 95% confidence interval [CI] 0.58-0.98; *I*^2^ = 0%) [26]. Our result was consistent with a cost-utility and budget impact analysis, which estimates that levalbuterol’s NDRL entry could save ¥22.3 million for hospitalized COPD patients in three years [27]. However, the results of another population-based retrospective study including 8,172 asthmatic patients aged 2~18 years demonstrated that levalbuterol was not associated with reduced hospitalizations (adjusted incidence ratio 0.93; 95% CI 0.99-1.63)[11]. Given the conflicting results above, this BIM considered no clinical benefit as a conservative estimate in the base-case analysis, and there was still a budget savings of ¥17.39 million.

The primary advantage of our research is that it is the first economic assessment carried out in China that focuses on the costs of nebulized SABA and examines the financial impact of levalbuterol in pediatric asthma. This information can help Chinese decision-makers allocate resources more effectively and enhance patient outcomes.

However, there are still several limitations of our study. First, a predictive analysis was required, so several input parameters, including the proportion of members potentially eligible for levalbuterol, albuterol, and terbutaline and the prevalence of asthma and the proportion of children treated with nebulized SABAs for asthma, needed to be predicted, and applied. These values had to be estimated from historical data [19,28,29]. Moreover, a few assumptions were made for usage data expectations regarding market share due to the nature of the IMS sales data. The IMS sales data used in the model did not have a further breakdown by disease and did not include the sales data from private hospitals; thus, the model assumed that the disease-specific breakdown would not significantly affect the market share of SABAs and would not change the results. Additionally, based on previous research, private hospitals account for only 0.3% of all hospitals providing pediatric services, with public hospitals comprising 99.7%. Therefore, the absence of private hospital data in the IMS sales data is not expected to significantly impact the results of our study [30].

## Conclusions

In conclusion, base-case analysis findings from the BIM suggest a potential for a moderate net budgetary effect when levalbuterol is used in pediatric asthma treatment, reflecting a downturn in healthcare spending. For now, the results of this budget impact analysis should be viewed as conservative estimates. BIM should be taken into consideration and evaluated when more information on assumption-based inputs becomes available.

## Appendices

**Table 8:** The CHEERS 2022 checklist.

Section/topic	Item No	Guidance for reporting	Reported in section
Title			
Title	1	Identify the study as an economic evaluation and specify the interventions being compared.	Title
Abstract			
Abstract	2	Provide a structured summary that highlights context, key methods, results, and alternative analyses.	Abstract
Introduction			
Background and objectives	3	Give the context for the study, the study question, and its practical relevance for decision making in policy or practice.	Introduction
Methods			
Health economic analysis plan	4	Indicate whether a health economic analysis plan was developed and where available.	Model Description and Structure
Study population	5	Describe characteristics of the study population (such as age range, demographics, socioeconomic, or clinical characteristics).	Population, Table 1
Setting and location	6	Provide relevant contextual information that may influence findings.	Population
Comparators	7	Describe the interventions or strategies being compared and why chosen.	Treatment Comparators and Market Share
Perspective	8	State the perspective(s) adopted by the study and why chosen.	Model Description and Structure
Time horizon	9	State the time horizon for the study and why appropriate.	Model Description and Structure
Discount rate	10	Report the discount rate(s) and reason chosen.	Model Description and Structure
Selection of outcomes	11	Describe what outcomes were used as the measure(s) of benefit(s) and harm(s).	Assumptions and Clinical Inputs, Model Outputs for the Base-Case Model
Measurement of outcomes	12	Describe how outcomes used to capture benefit(s) and harm(s) were measured.	Model Outputs for the Base-Case Model
Valuation of outcomes	13	Describe the population and methods used to measure and value outcomes.	Population, Assumptions and Clinical Inputs, Cost Inputs
Measurement and valuation of resources and costs	14	Describe how costs were valued.	Cost Inputs, Model Outputs for Base Case Model
Currency, price date, and conversion	15	Report the dates of the estimated resource quantities and unit costs, plus the currency and year of conversion.	Model Description and Structure
Rationale and description of model	16	If modelling is used, describe in detail and why used. Report if the model is publicly available and where it can be accessed.	Model Description and Structure, Assumptions and Clinical Inputs
Analytics and assumptions	17	Describe any methods for analysing or statistically transforming data, any extrapolation methods, and approaches for validating any model used.	Statistical Analysis, Assumptions and Clinical Inputs
Characterising heterogeneity	18	Describe any methods used for estimating how the results of the study vary for subgroups.	Scenario and Sensitivity analysis
Characterising distributional effects	19	Describe how impacts are distributed across different individuals or adjustments made to reflect priority populations.	One-way Sensitivity Analysis
Characterising uncertainty	20	Describe methods to characterise any sources of uncertainty in the analysis.	Scenario and Sensitivity analysis
Approach to engagement with patients and others affected by the study	21	Describe any approaches to engage patients or service recipients, the general public, communities, or stakeholders (such as clinicians or payers) in the design of the study.	Not applicable
Results			
Study parameters	22	Report all analytic inputs (such as values, ranges, references) including uncertainty or distributional assumptions.	Results
Summary of main results	23	Report the mean values for the main categories of costs and outcomes of interest and summarise them in the most appropriate overall measure	The base-case analysis
Effect of uncertainty	24	Describe how uncertainty about analytic judgments, inputs, or projections affect findings. Report the effect of choice of discount rate and time horizon, if applicable.	Scenario and Sensitivity analysis
Effect of engagement with patients and others affected by the study	25	Report on any difference patient/service recipient, general public, community, or stakeholder involvement made to the approach or findings of the study.	Not applicable
Discussion			
Study findings, limitations, generalisability, and current knowledge	26	Report key findings, limitations, ethical or equity considerations not captured, and how these could affect patients, policy, or practice.	Discussions
Other relevant information			
Source of funding	27	Describe how the study was funded and any role of the funder in the identification, design, conduct, and reporting of the analysis	Acknowledgments
Conflicts of interest	28	Report authors conflicts of interest according to journal or International Committee of Medical Journal Editors requirements.	Conflicts of Interest
